# Investigation of Oxidation Methods for Waste Soy Sauce Treatment

**DOI:** 10.3390/ijerph14101190

**Published:** 2017-10-07

**Authors:** Hyun-Hee Jang, Gyu-Tae Seo, Dae-Woon Jeong

**Affiliations:** 1 Department of Environmental and Chemical Engineering, Eco-Friendly Offshore Plant FEED Engineering Course, Changwon National University, 20 Changwondaehak-ro, Uichang-gu, Changwon-si, Gyeongsangnam-do 51140, Korea; jang_@changwon.ac.kr; 2 School of Civil, Environmental and Chemical Engineering, Changwon National University, 20 Changwondaehak-ro, Uichang-gu, Changwon-si, Gyeongsangnam-do 51140, Korea

**Keywords:** waste soy sauce, color removal, COD lowering, fenton, fenton-like, ozone

## Abstract

To obtain a suitable oxidation method for removing the color and lowering the chemical oxygen demand (COD) of waste soy sauce, Fenton (Fe^2+^), Fenton-like (Fe^3+^), and ozone (O_3_) oxidation methods are used as the target reactions. In experimental conditions for Fenton oxidation, the dose of Fe^2+^ and Fe^3+^ was varied between 100 mg/L and 300 mg/L. The dose of hydrogen peroxide for the reaction was injected from 100–1000 mg/L. For ozone oxidation, the pH was increased from 3 to 14 and the O_3_-containing gas was supplied continuously for 30 min through a gas diffuser at the bottom of the reactor at different applied O_3_ doses (10–90 mg/L). We subjected it to a simple 1:20 dilution with deionized water to identify the comparison result in detail. O_3_ oxidation shows the highest efficiencies of color removal (81.1%) and COD lowering (64.9%) among the three oxidation methods. This is mainly due to the fact that it has a relatively large amount of hydroxyl radical, resulting in the degradation of organics. Thus, O_3_ oxidation could be a promising method for removing the color and lowering the COD of waste soy sauce. The critical parameters (pH and applied O_3_ dose) were varied systematically to optimize O_3_ oxidation. It was found that the optimum pH and applied O_3_ dose are 11.0 mg /L and 50.0 mg /L, respectively (color removal = 34.2%, COD removal = 27.4%).

## 1. Introduction

Soy sauce is an oriental product that is used as a condiment or as a coloring agent in food preparation and is consumed widely in Asian countries such as Korea, China, Japan, Thailand, Indonesia, and Malaysia. In Korea, specifically, the amount of waste soy sauce that is generated increased rapidly from 30,200 tons to 61,200 tons over 3 years (2011 to 2013) [1]. Ocean dumping of this waste is prohibited by the amended London Dumping Convention, and it is treated instead. However, the cost of treating waste soy sauce is $300 per ton, which is much more than common wastewater treatment costs. For this reason, many researchers have tried to develop economic treatment methods for waste soy sauce.

Soy sauce wastewater has shock load changes, there are seasonal changes in the water quality, and the quality of wastewater fluctuates considerably. The contamination component of soy sauce wastewater is not stable, and soy sauce factories make many kinds of products; therefore, the composition of soy sauce wastewater is very complex [2,3,4]. This is because soy sauce is produced through a long period of fermentation, and thus contains a high organic chemical oxygen demand (COD) value and a deep dark brown color due to the caramel pigment and Maillard reaction (melanin or melanoidin) [5,6].

In the last few years, the possibility of eliminating the color and COD of soy sauce wastewater has been intensively investigated in an effort to overcome the limits of biodegradation treatments [7,8,9].

Zheng L. et al. reported that sludge reuse in coagulation is a cost-effective method for soy sauce wastewater treatment [3]. Likewise, some researchers reported that more improvements in the removal efficiency of biodegradation treatment were achieved by demonstrating the sequential batch reactor (SBR) system [7,8]. Zuo J. et al. reported that the COD removal rate of soy sauce wastewater reached 86.2% using the SBR process [8]. Futhermore, to meet discharge limits, coagulation and oxidation following the use of a membrane bio–reactor (MBR) were verified as effective for reducing the residual color and COD [9]. Nevertheless, there are still some technological challenges to be solved. Thus, oxidation treatment has been considered as one of the alternative treatment method for soy sauce wastewater. Kai S. et al. reported that research on the application of Fenton oxidation to remove the caramel color of sauce wastewater [2]. However, only a limited number of studies have been concerned with this oxidation treatment. Futhermore, waste soy sauce is more colored and has higher COD compared to wastewater from livestock or industries. It is well known that biological treatment can reduce COD levels effectively to less than about 2000 mg/L in treatment plants for wastewater with high organic concentration [10,11]. However, conventional biological treatment is not suitable for waste soy sauce because of the latter’s very high salinity of 168.2‰. Current research on evaluation methods for assessing these treatment technologies is scattered and lacks valid and integrated evaluation methods for assessing the treatment effectiveness [12]. Hence, it is necessary to develop an advanced treatment method for waste soy sauce that can recover the salt and lower the COD simultaneously.

In recent decades, there has been much interest in oxidation reactions because of their elevated ability to remove color and lower COD. In addition, oxidation reactions are expected to recover high concentrations of salt. Therefore, many attempts have been made to develop an oxidation method based on the hydroxyl radical that could overcome the limitations of biological treatment [13,14,15,16]. Among the various reported oxidants, Fenton’s reagent (FeSO_4_∙7H_2_O, Fe^2+^), a Fenton-like reagent (FeO(OH), Fe^3+^), and ozone (O_3_) are the most suitable for treating high color and COD.

For the color/COD treatment of livestock wastewater by the Fenton method (Fe^2+^/H_2_O_2_), Lee and Shoda (2008) reported removal efficiencies of 88.0% and 95.4% for the color and COD of the supernatant, respectively, after static precipitation of the produced sludge [14]. Solmaz and Birgül (2006) showed that Fenton-like oxidation (Fe^3+^/H_2_O_2_) could be used to remove color and lower COD in biologically pretreated textile wastewater with efficiencies of 71.0% and 64.0%, respectively [15]. Yang and Yuan (2016) recorded a maximum COD-lowering efficiency of 92.5% for O_3_ oxidation, with the biodegradability index of the dye effluent increasing from an initial value of 0.18 to 0.49 [16]. According to the aforementioned results, O_3_ oxidation is the preferred removal method. However, despite research progress to date, there has been no optimization of oxidation methods for the removal of the strong color of waste soy sauce and the lowering of its high COD.

In the present study, we compare the color-removal and COD-lowering efficiencies of the Fenton (FeSO_4_∙7H_2_O, Fe^2+^), Fenton-like (FeO(OH), Fe^3+^), and ozone (O_3_) oxidation method and try to explain their differences. In particular, the operating conditions (pH and applied O_3_ dose) of the O_3_ oxidation method are changed systematically for optimization. We characterize the effects of pH and the applied O_3_ dose on the oxidation environment, and we relate the characterization results to the removal/lowering efficiency for waste soy sauce.

## 2. Materials and Methods

### 2.1. Sampling Procedure

The soy sauce used in this study was provided by Monggo Foods, Inc., Changwon-si, Korea. We refer to it simply as soy sauce if it remains within its expiration date, or waste soy sauce otherwise. Because of the high concentration of organic matter in waste soy sauce, we subjected it to a simple 1:20 dilution with deionized water to identify the result in detail. However, in the optimization experiments, samples of waste soy sauce were used without dilution.

### 2.2. Characterization

We used a Seven Compact S220 pH/ion meter for the pH measurements. The concentrations of biochemical oxygen demand (BOD), chemical oxygen demand (COD_cr_), total nitrogen (T–N), and total phosphorus (T–P) were measured using the Hach^CO^ US/DR3900 (320–1100 nm) kit assay method. All these characterizations were analyzed using a method for testing water pollution (Ministry of Environment, Korea). Total organic carbon (TOC) was analyzed using a Shimadzu JP/TOC–5000A analyzer (Kyoto, Japan), and the color was calculated as the transmittance per 10 wavelengths using an Agilent Cary60 UV–VIS spectrophotometer (Santa Clara, CA, Untied States). The salinity was measured using an SB1500PRO (0–10%, HM Digital, Seoul, Korea) portable salinometer, and all samples were analyzed with their impurities removed by a 1.2 μm–GF/C (Whatman) filter.

### 2.3. Fenton Oxidation

Table 1 summarizes the experimental conditions for Fenton (Fe^2+^, Fe^3+^) oxidation. A jar test was used to mix the sample and the oxidant. A stock 10-g/L solution of Fe^2+^ was prepared by dissolving FeSO_4_·7H_2_O (Fe^2+^) and FeO(OH) (Fe^3+^) (Sigma-Aldrich, Seoul, Korea) in 0.2 N H_2_SO_4_. In addition to the iron sulfate reagent, a 34.5% H_2_O_2_ solution (7722–84–1, Samchun Pure Chemicals, Korea) was also used. Experimental runs were performed in 1-L glass beakers with 0.5 L of sample. First, the pH of the sample was adjusted to 3.0 (Yetilmezsoy and Sakar, 2008) by adding 1 N H_2_SO_4_ [13]. The Fe^2+^ and H_2_O_2_ solutions were then added to the waste soy sauce, which was stirred rapidly for 10 min at 150 rpm using jar-test equipment (Chang Shin Science Co., Seoul, Korea). The waste soy sauce was then stirred at 30 rpm for a further 20 min, after which any flocs that formed were allowed to settle for 1 h. About 50 mL of the supernatant sample was then collected for color and COD determination. To evaluate the Fenton method, different experimental runs were performed. First, the initial pH, the H_2_O_2_ dose, and the reaction time were kept constant while the dose of Fe^2+^ and Fe^3+^ was varied between 100 mg/L and 300 mg/L. Second, the initial pH, the dose of Fe^2+^ and Fe^3+^, and the reaction time were kept constant while the H_2_O_2_ dose was varied between 100 mg/L and 1000 mg/L. All experimental runs were conducted in duplicate, and the results are expressed as mean values.

### 2.4. Ozone (O_3_) Oxidation

**Figure 1:**
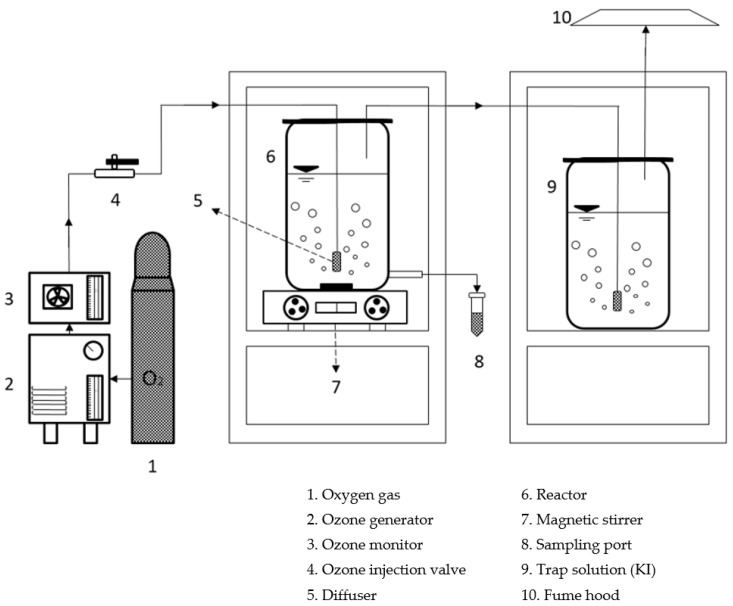
Schematic diagram of the O_3_-oxidation experimental system.

A schematic of the bench-scale reactor system used for the experiment throughout the study is shown in Figure 1. Ozone (O_3_) experiments were conducted in a 1-L Pyrex reactor connected to an O_3_ generator (LAB–I, Ozone Tech, Daejeon, Korea) that produced O_3_ from oxygen by electrical discharge. Table 2 shows the experimental conditions for O_3_ oxidation. The reactor was filled with 0.5 L of waste soy sauce and then agitated with a magnetic stirrer at 150 rpm. The change of color and the lowering of COD were observed while the pH was increased from 3 to 14. The O_3_-containing gas was supplied continuously for 30 min through a gas diffuser at the bottom of the reactor at different applied O_3_ doses (10–90 mg/L). Two O_3_ traps containing 2% potassium iodide solution were connected in series with the reactor to confirm the vented O_3_ gas concentration in the outlet gas stream, and 0.1 N Na_2_S_2_O_3_ (sodium thiosulfate) was used as the reducing agent for the reverse titration in the trap. At the time of O_3_ reaction with the solution, 0.1 N H_2_SO_4_ (sulfuric acid) was used to maximize the reaction of the O_3_ in the liquid phase to the I_2_ state. Sample aliquots (10 mL) were taken from the reactor at regular intervals for color and COD determination. All these experimental runs were performed in duplicate at the various pH values of the waste soy sauce (pH 3–14) and at room temperature in a fume hood for safety from O_3_ gas. The applied O_3_ dose in the O_3_ oxidation reaction and the concentration of discharged O_3_ gas in the atmosphere phase after the reaction were calculated using Equation below:

$$\frac{\left(①\right)eqNa_{2}S_{2}O_{3}}{L}\frac{1eqO_{3}}{2eqNa_{2}S_{2}O_{3}}\frac{48gO_{3}}{1eqO_{3}}\frac{\left(②\right)\mathrm{mL}}{\left(③\right)\min}\frac{1}{Reactor\;volume\left(L\right)}=\left(④\right)\mathrm{mg}O_{3}/L\cdot \min$$

① Concentration of Na_2_S_2_O_3_ prepared according to the concentration of O_3_ produced
② Capacity of Na_2_S_2_O_3_ consumed in the inverse titration (mL)
③ Time over which the O_3_ gas was captured (min)
④ Applied O_3_ dose (mg/L·min)

## 3. Results and Discussion

### 3.1. Characterization of Waste Soy Sauce

To verify the properties of waste soy sauce, we analyzed it using a method for testing water pollution (Ministry of Environment, Republic of Korea) and instrumental analysis. Table 3 summarizes the characteristics of soy sauce and waste soy sauce. The colors were 3904 and 4038 TCU, respectively, and the COD concentrations were 228.3 g/L and 229.1 g/L, respectively. Both the quantities are lower for soy sauce than those for waste soy sauce, indicating that the color and COD concentration increased by expiry (i.e., when soy sauce becomes waste soy sauce). However, the differences between the characteristics are not considerable. In comparison, the color and COD concentration of livestock wastewater ranges are 150–450 TCU and 10–50 g/L [17,18,19]. Therefore, we have confirmed that waste soy sauce has high color value and COD concentration.

To confirm the detailed properties of waste soy sauce, the characteristics of each experiment were analyzed five times for accuracy. Table 4 shows the experimental results for COD_cr_, BOD, T–N, T–P, TOC, salinity, and color. There were no major differences in the characteristics of the five samples from the experimental results. Given its pH value of 4.5, waste soy sauce is acidic. In addition, the T–N and T–P analysis results were 9.8 g/L and 3.1 g/L, respectively, which indicates that waste soy sauce is similar to livestock wastewater in those characteristics [20,21]. The salinity of waste soy sauce was 168.2‰, which is much higher than that of food waste (35.0‰) [22,23]. Interestingly, the color, BOD, and COD concentrations of waste soy sauce were 3948 TCU, 129.0 g/L, and 231.6 g/L, which are incomparably higher than the respective values for livestock wastewater (color = 150–450 TCU, COD = 10–50 g/L) [11,14,18,21]. It can be concluded that wastewater with high color value and COD concentration is difficult to treat by common treatment methods.

Thus, it is necessary to develop an advanced oxidation method for waste soy sauce. For this reason, three types of oxidation method used to lower the color and COD values were applied to waste soy sauce, and their efficiencies were compared. In addition, we also attempted to determine the optimum pH value and applied O_3_ dose that resulted in the maximum oxygenation capacity in O_3_ oxidation.

### 3.2. Reaction Results by Oxidation

**Figure 2:**
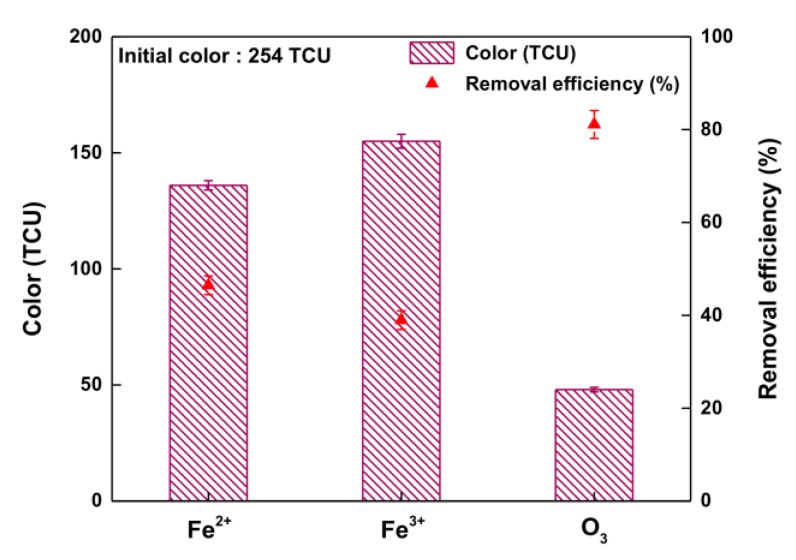
Removal efficiency of color of Fenton (Fe^2+^), Fenton-like (Fe^3+^), and O_3_ oxidation.

Figure 2 shows the color-removal efficiencies of Fenton (Fe^2+^), Fenton-like (Fe^3+^), and ozone (O_3_) oxidation. The initial color of the waste soy sauce was 254.0 TCU. With regard to the final color after oxidation, Fenton-like oxidation (Fe^3+^) showed the highest color value of 155.0 TCU, whereas O_3_ oxidation showed the lowest color value of 48.0 TCU. As a result, the color-removal efficiency decreased in the following order: 81.1% (O_3_) > 46.5% (Fe^2+^) > 38.9% (Fe^3+^). It is well known that the oxidant O_3_ is highly capable of oxidation from the hydroxyl radical [24,25,26]. Among the examined oxidation methods, O_3_ oxidation can be selected as the most promising treatment method for waste soy sauce on the basis of its relatively high color-removal efficiency.

**Figure 3:**
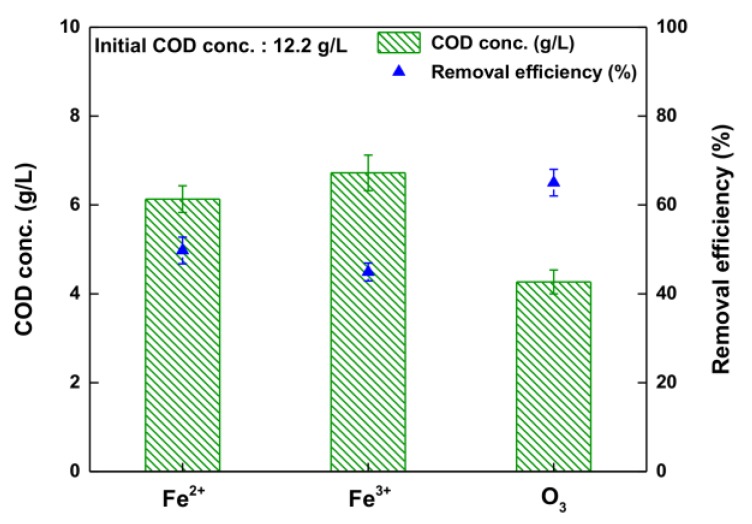
Removal efficiency of COD of Fenton (Fe^2+^), Fenton-like (Fe^3+^), and O_3_ oxidation.

Figure 3 shows the COD-lowering efficiencies of the different oxidation methods. The initial COD concentration of the waste soy sauce was 12.2 g/L (although the sample had been diluted with deionized water, 50 mL of waste soy sauce per L of DI water). Subsequently, the COD concentration was reduced to 6.1 g/L (Fe^2+^), 6.7 g/L (Fe^3+^), and 4.3 g/L (O_3_) from its initial value (12.2 g/L). In other words, the COD concentration reduced by 49.7% (Fe^2+^), 44.9% (Fe^3+^), and 65.0% (O_3_). Again, it should be noted that O_3_ oxidation showed the highest reduction in COD among the various oxidation methods. This is because O_3_ oxidant is much more efficient than the others (Fe^2+^ or Fe^3+^) at treating waste soy sauce. Thus, we selected the O_3_ oxidation method. Although O_3_ oxidation by hydroxyl radical shows great promise as a way to lower the COD of waste soy sauce, its potential has not been studied systematically. Therefore, optimization of O_3_ oxidation is needed for waste soy sauce treatment that can remove color and lower COD simultaneously.

### 3.3. Optimization of Parameters in Ozone (O_3_) Oxidation

#### 3.3.1. Effect of pH

It is well known that pH can affect the stability of O_3_. The pH of water is important in freshly ozonated water because hydroxide ions initiate O_3_ decay by a reaction with substances. At high pH, O_3_ decay is faster than that at low pH. The stability of O_3_ depends largely on the water matrix, particularly the pH, water temperature, natural organic matter content, and alkalinity [25]. The pH of raw water is influential because hydroxide ions initiate O_3_ decomposition, and decomposition reactions can be accelerated artificially by increasing the pH [26]. To optimize the pH, we changed it systematically.

**Figure 4:**
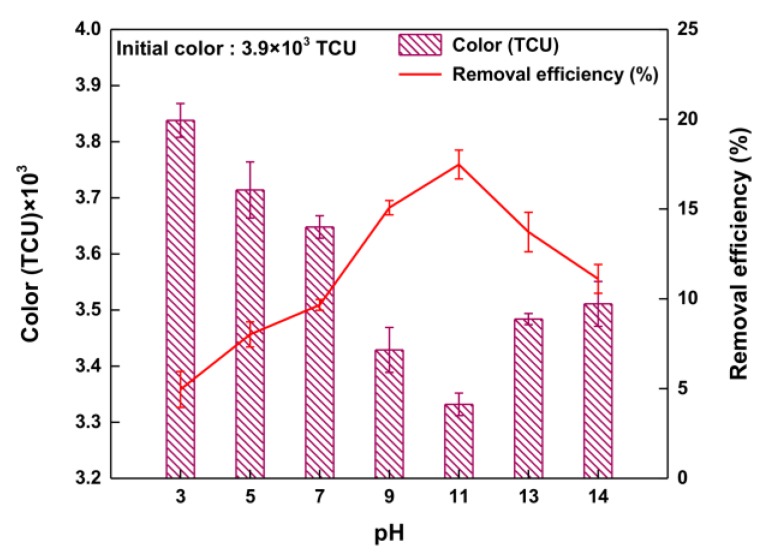
Removal efficiency of color with various pH values during O_3_ oxidation.

The effect of pH on color removal by O_3_ oxidation in waste soy sauce was investigated at pH of 3, 5, 7, 9, 11 and 13 (applied O_3_ dose = 10 mg/L). The efficiency of color removal from waste soy sauce with various pH levels is shown in Figure 4. According to the reaction results, the color-removal efficiency changed dramatically with pH value. At pH 3.0, the color-removal efficiency was 4.9%, increasing significantly to 17.5% when the pH was increased to 11.0. The color-removal efficiency at pH 11.0 was the highest value, mainly because of an increased amount of hydroxyl radical [27]. Above pH 11.0, the color-removal efficiency clearly decreased (from 17.5% to 11.1%), mainly because oxidation above pH 11.0 has a strong scavenging effect of carbonate (CO_3_^2−^) and bicarbonate (HCO^3−^), resulting in lower color-removal efficiency [13,16,24]. This result shows clearly that the color-removal efficiency depends strongly on the pH of the oxidation method [25,26]. As a result, an optimum pH is found to be 11.0 during the O_3_ oxidation reaction because of the high oxygenation capacity resulting from a suitable amount of hydroxyl radical.

**Figure 5:**
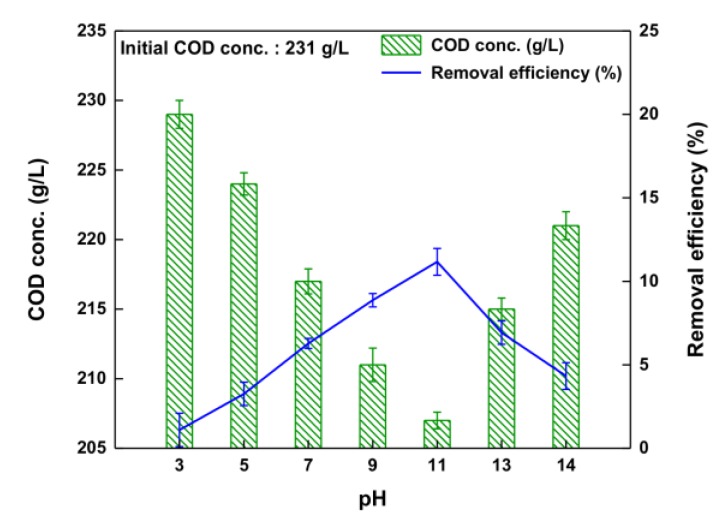
Removal efficiency of COD with various pH values during O_3_ oxidation.

**Figure 6:**
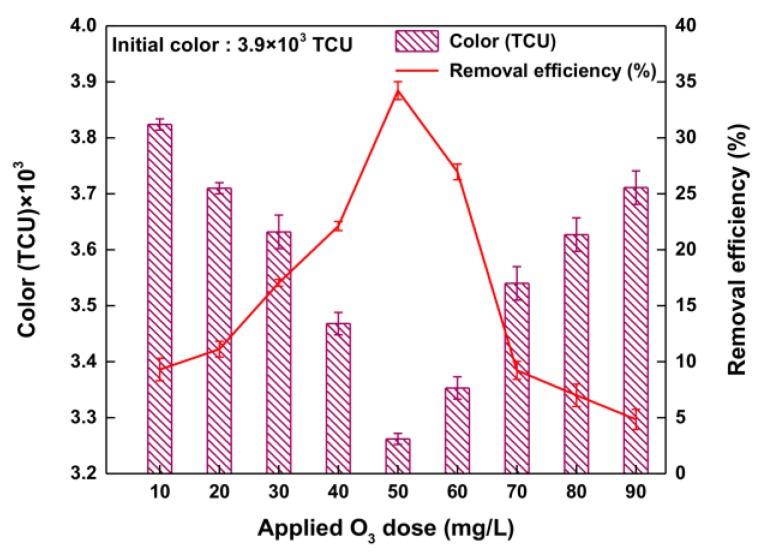
Removal efficiency of color with various applied O_3_ doses for O_3_ oxidation.

Figure 5 shows the efficiency of lowering the COD of the waste soy sauce as a function of pH. The effect of pH on lowering COD by O_3_ oxidation was evaluated at pH from 3.0 to 14.0 with a dose of 10 mg/L of O_3_ applied to the waste soy sauce. According to the reaction results, the trend in COD-lowering efficiency is similar to that of color-removal efficiency shown in Figure 5. As the pH was increased from 3.0 to 11.0, the COD-lowering efficiency increased gradually from 1.1% to 11.2%. Consequently, the COD-lowering efficiency was the highest at pH 11.0. In other words, the COD was lowered by 24 g/L at pH 11 during O_3_ oxidation from an initial value of 231 g/L. This indicates that the amount of hydroxyl radical was increased sufficiently by increasing the pH [20]. The COD-lowering efficiency decreased distinctly (from 11.2% to 4.3%) when the pH exceeded 11.0. We conclude that hydroxyl radicals are scavenged by carbonate (CO_3_^2−^) and bicarbonate (HCO^3−^) at pH values above 11.0 [24,28]. The results shown in Figure 5 and Figure 6 confirm that the color-removal and COD-lowering efficiencies are highly dependent on pH, with the highest values at pH 11.0.

#### 3.3.2. Effect of Ozone (O_3_) Dose

To optimize the applied O_3_ dose, we changed it systematically. Kwon et al. [24] reported that increasing the applied O_3_ dose should enhance mass transfer and cause an increased O_3_ dose in the liquid phase and an increased pseudo-first-order rate constant [29,30]. In the O_3_ oxidation experiment, the applied O_3_ dose is a key parameter determining the removal effect [25]. In detail, O_3_ injection increases the formation of hydroxyl radicals, which are highly effective for destroying the structure of organic matter in wastewater [24]. To investigate the effect of the applied O_3_ dose on the removal efficiency, we conducted experiments with various O_3_ doses (10, 20, 30, 40, 50, 60, 70, 80, and 90 mg/L) at pH 11.0.

Figure 6 shows the reaction results of color-removal efficiency for various applied O_3_ doses. The reaction results show that the color-removal efficiency changes considerably with increasing O_3_ dose [31]. At an applied O_3_ dose of 10.0 mg/L, the color-removal efficiency was 9.3%, and this increased significantly to 34.2% when the applied O_3_ dose was increased to 50.0 mg/L. Within the applied O_3_ dose range of 10.0–50.0 mg/L, the color-removal efficiency increased from 11.1% at 20.0 mg/L to 34.2% at 50.0 mg/L. This suggests that hydroxyl radicals play an active role in the O_3_ oxidation reaction to attain a high color-removal efficiency; the color-removal efficiency was highest at an applied O_3_ dose of 50.0 mg/L. During O_3_ oxidation above 50.0 mg/L, however, even with the higher O_3_ dose provided, the color-removal efficiency decreased [25,31]. This was attributed mainly to the fact that O_3_ oxidation at an applied O_3_ dose above 50.0 mg/L involves a large amount of hydroxyl radical reacting instantaneously with O_3_-demanding species (target substances). In other words, there is a high chance of instantaneous ozone demand in the waste soy sauce [32,33]. This result indicates that the applied O_3_ dose in the oxidation reaction affects the amount of hydroxyl radical, which can directly affect the removal efficiency [34]. Consequently, the optimum applied O_3_ dose is found to be 50.0 mg/L for the O_3_ oxidation reaction.

**Figure 7:**
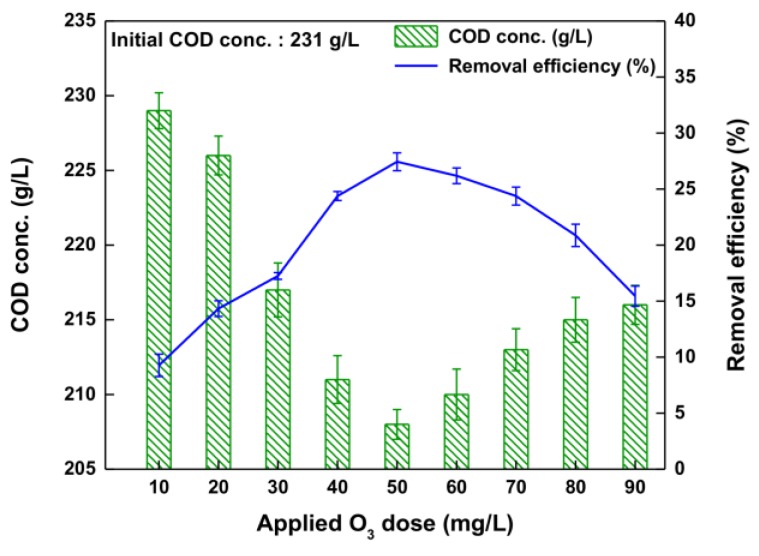
Removal efficiency of COD with various applied O_3_ doses for O_3_ oxidation.

Figure 7 shows the results of varying the COD-lowering efficiency by changing the applied O_3_ dose. To verify the effect of the applied O_3_ dose on lowering the COD of waste soy sauce by O_3_ oxidation, the applied O_3_ dose was varied from 10.0 mg/L to 90.0 mg/L. In addition, to optimize the applied O_3_ dose, the experiment was performed at the pH value of 11.0 obtained in the color-removal experiment. According to the O_3_ reaction results, the COD-lowering efficiency changed with the applied O_3_ dose. As the applied O_3_ dose was increased from 10.0 mg/L to 50.0 mg/L, the COD-lowering efficiency increased gradually from 9.3% to 27.4%. Consequently, the dose of 50.0 mg/L showed the highest value of COD-lowering efficiency. That is, the COD was lowered by 23.0 g/L at an applied O_3_ dose of 50.0 mg/L during the O_3_ oxidation from an initial value of 231.0 g/L. This result indicates that the amount of hydroxyl radical was increased sufficiently by increasing the applied O_3_ dose [35]. The COD-lowering efficiency decreased distinctly (from 27.4% to 15.5%) when the applied O_3_ dose exceeded 50.0 mg/L. This was due mainly to the fact that the hydroxyl radical was consumed instantaneously by O_3_-demanding species, even though the applied O_3_ dose was high [24,25,36]. Thus, it can be concluded that the COD-lowering efficiency was improved by increasing the applied O_3_ dose to 50.0 mg/L, whereas a further increase from 60.0 mg/L to 90.0 mg/L resulted in decreased efficiency. These results show clearly that the COD-lowering efficiency is highly dependent on the O_3_ dose [34,37]. All these results confirm that the optimum applied O_3_ dose to lower the COD of the waste soy sauce by O_3_-oxidation treatment is 50.0 mg/L.

## 4. Conclusions

Fenton (Fe^2+^), Fenton-like (Fe^3+^), and ozone (O_3_) oxidation methods were evaluated for removing the color and lowering the COD of waste soy sauce. Of these oxidation methods, O_3_ oxidation exhibited the highest color-removal and COD-lowering efficiencies. This was due, primarily, to the high oxidation capability of O_3_ by hydroxyl radical. Thus, O_3_ oxidation can be considered to be one of the best treatment methods for soy sauce, which requires high intrinsic color-removal and COD-lowering efficiencies. We also confirmed that the color-removal and COD-lowering efficiencies for waste soy sauce depend strongly on the pH, the amount of hydroxyl radical, and the applied O_3_ dose. The O_3_ oxidation of waste soy sauce under optimized parameter conditions (pH = 11.0, applied O_3_ dose = 50 mg/L) had the highest efficiencies of color removal (34.2%) and COD lowering (27.4%).

## Figures and Tables

**Table 1:** The experimental conditions for the Fenton (Fe^2+^, Fe^3+^) oxidation method.

Parameters	Value
pH	3.0
Dose of Fe^2+^ and Fe^3+^ (mg/L)	100, 150, 200, 250, 300
Dose of H_2_O_2_ (mg/L)	100, 200, 300, 400, 500, 600, 700, 800, 900, 1000
Mixing time (min)	30
Mixing speed (rpm)	150 (10 min) and 30 (20 min)
Settling time (min)	60
Sample volume (mL)	500

**Table 2:** The experimental conditions for O_3_-oxidation method.

Parameters	Value
pH	3.0, 5.0, 7.0, 9.0, 11.0, 13.0, 14.0
Dose of O_3_ (mg/L)	10, 20, 30, 40, 50, 60, 70, 80, 90
Mixing time (min)	30
Mixing speed (rpm)	150
Sample volume (mL)	500

**Table 3:** Chemical composition of soy sauce and waste soy sauce.

Parameters	Soy Sauce	Waste Soy Sauce
pH	4.44	4.66
COD (g/L)	228.3	229.1
BOD (g/L)	142.5	123.6
T−N (g/L)	10.0	10.9
T−P (g/L)	2.9	2.5
TOC (g/L)	60.5	56.6
Salinity (‰)	166	154
Color (TCU)	3904	4038

**Table 4:** Characteristics of waste soy sauce.

Division	pH	COD (g/L)	BOD (g/L)	T−N (g/L)	T−P (g/L)	TOC (g/L)	Salinity (‰)	Color (TCU)
Sample Ⅰ	4.6	231.5	129.4	10.4	2.8	57.6	167.0	3918
Sample Ⅱ	4.4	228.4	123.3	8.9	3.3	60.6	174.0	4024
Sample Ⅲ	4.7	233.8	131.5	9.4	2.7	61.4	191.0	3905
Sample Ⅳ	4.3	235.3	130.8	9.9	3.1	62.0	153.0	4008
Sample Ⅴ	4.6	228.8	129.9	10.6	3.6	58.5	156.0	3884
Average	4.5	231.6	129.0	9.8	3.1	60.0	168.2	3948
